# Hemisphere opposite to vascular trunk deviation is earlier affected by glaucomatous damage in myopic high-tension glaucoma

**DOI:** 10.1371/journal.pone.0233270

**Published:** 2020-05-18

**Authors:** Kyoung Min Lee, Martha Kim, Sohee Oh, Seok Hwan Kim

**Affiliations:** 1 Department of Ophthalmology, Seoul National University College of Medicine, Seoul, Korea; 2 Department of Ophthalmology, Seoul National University Boramae Medical Center, Seoul, Korea; 3 Department of Ophthalmology, Dongguk University Ilsan Hospital, Goyang, Korea; 4 Department of Biostatistics, Seoul National University Boramae Medical Center, Seoul, Korea; University of California San Diego, UNITED STATES

## Abstract

**Purpose:**

To investigate whether the position of the central vascular trunk, as a surrogate of lamina cribrosa (LC) shift, is associated with the initial hemisphere of visual field defect in myopic high-tension glaucoma (HTG) eyes.

**Methods:**

The deviation of the central vascular trunk was measured from the center of the Bruch’s membrane opening (BMO), which was delineated by OCT imaging. The angular deviation was measured with the horizontal nasal midline as 0° and the superior location as a positive value. The initial hemisphere developing visual field defect was defined as three connected abnormal points (having a *P* value with less than 0.5% probability of being normal) appearing in only one hemisphere in pattern deviation plots. If those points were observed in both hemispheres initially, the eye was classified as bi-hemispheric visual field defect.

**Results:**

Initially, 36 eyes (44%) had superior visual field defects, 27 (33%) inferior visual field defects, and 18 (22%) bi-hemispheric visual field defects. After a mean follow-up of 5 years, the number of bi-hemispheric visual field defects had increased to 34 (42%). A logistic regression analysis revealed that inferior deviation of vascular trunk was the only factor associated with initial inferior visual field defect (*P* = 0.001), while initial bi-hemispheric visual field defects were associated with worse mean deviation at initial visits (*P*<0.001). A conditional inference tree analysis showed that both the angular deviation (*P*<0.001) and initial mean deviation (*P* = 0.025) determined the initial hemispheres developing visual field defect.

**Conclusions:**

Although both hemispheres were involved as glaucoma progression, the axons on the side counter to the vascular trunk deviation were damaged earlier in HTG. This finding implies the LC shift could add additional stress to axons exposed to high intraocular pressure.

## Introduction

Glaucoma is a progressive optic neuropathy that is characterized by selective loss of retinal ganglion cells [1–3]. The axons of retinal ganglion cells are thought to be damaged in the lamina cribrosa (LC), the morphology and regional differences of which are closely related to the severity and area susceptible to glaucomatous optic neuropathy [4–6]. The axonal insult is induced by intraocular pressure (IOP)-related stress and strain loaded onto the connective tissue of the optic nerve head (ONH), which includes the LC [7]. However, the strain exerted on the LC is not dependent purely on the IOP.

In the recent Boramae Myopia Cohort Study, we found that the inner retinal structure of the posterior polar area including the Bruch’s membrane opening (BMO) was relatively preserved during axial elongation, while the outer load-bearing sclera expanded [8–10]. This expansion resulted in the shift of the sclera and LC, which may be suggestive of another source of tensile stress exerted on the LC in myopic eyes. This speculation was supported by our subsequent study: in myopic normal-tension glaucoma (NTG), the location of glaucomatous damage has been found to be highly dependent on the direction of vascular trunk deviation as a surrogate of LC shift [11]. To summarize, LC shift might make some part of the LC more susceptible to damage even within the normal range of IOP [11]. This could also explain why some myopic eyes do not progress for several years without treatment [12]. Once the vulnerable pores in the direction opposite to LC shift have been severely damaged, glaucoma might not progress, since the pores in the other area would be relatively spared from the LC-shift-associated tensile stress [11].

**Fig 1:**
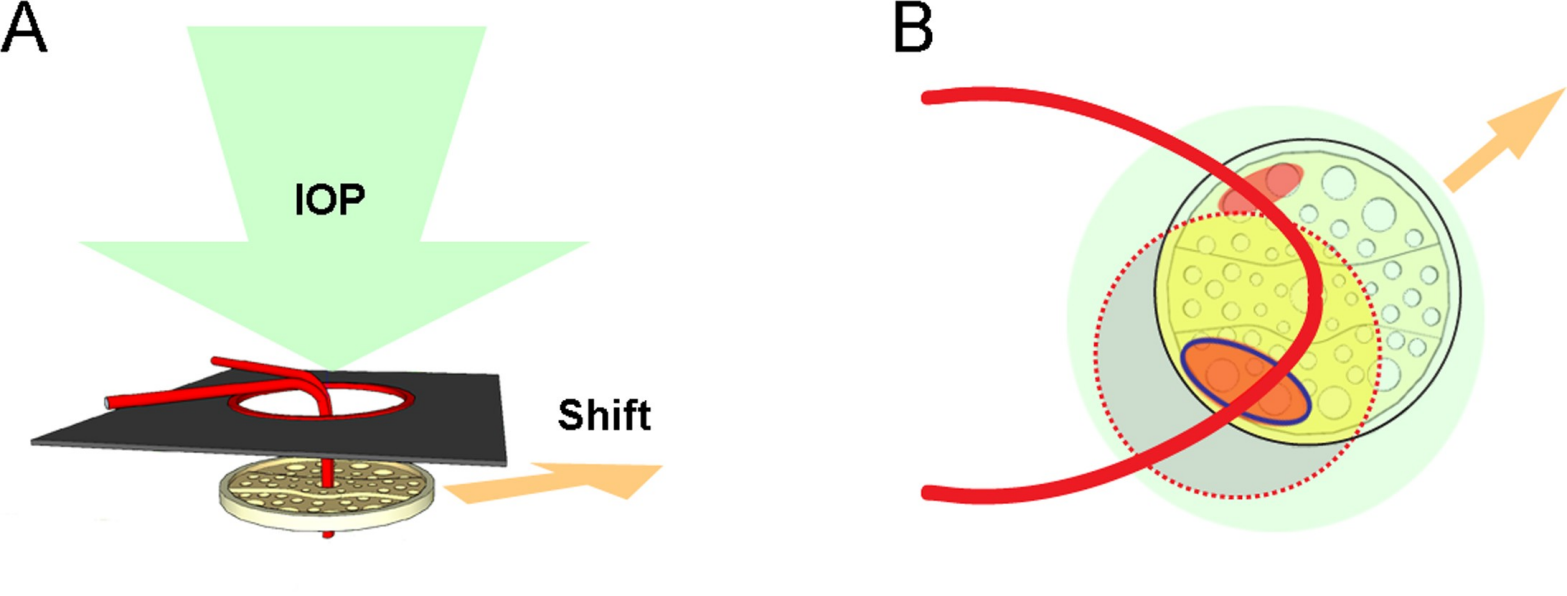
Effects of high intraocular pressure (IOP) and lamina cribrosa (LC) shift in high-tension glaucoma. (**A**) IOP can exert uniform force over the LC (green arrow). (**B**) The en face view. The LC shift (orange arrow) would make the pores on the side opposite to the shift more susceptible to IOP and glaucomatous damage. The red-dotted circles indicate the BMO margin, the black-solid circle indicates the LC, the portions in yellow color represent the visible part of the LC via funduscopic examination (clinical disc margin), and the red ellipsoids indicate the superotemporal and inferotemporal pores that are more susceptible to glaucomatous damage. Both pores would be affected by IOP (green circle), while the pores on the side opposite to the shift (the red ellipsoid marked with blue line) would be doubly affected by IOP (green circle) and shift-associated tensile stress (orange arrow).

In high-tension glaucoma (HTG), however, the ONH would be exposed to the radical stress of high IOP (Fig 1). Therefore, those myopic eyes might suffer doubly from LC-shift-associated tensile stress and high IOP. This speculation is supported by a previous study showing more rapid glaucoma progression in myopic eyes than in non-myopic eyes in patients with uncontrolled IOP over 21mmHg [13]. Based on that finding, we postulated that if the LC shift increases the vulnerability of the ONH, the more deformed and vulnerable pores might be affected earlier and the lesser deformed and relatively healthy pores might be affected later. This could explain the differences between the natural courses of HTG and NTG in myopic eyes. Therefore, the purpose of the present study was to determine, by observing the initial hemisphere of visual field development and the vascular trunk deviation, if the LC shift might be associated with ONH vulnerability in myopic HTG.

## Methods

This investigation was based on myopic HTG patients included in the Boramae Glaucoma Imaging Study (BGIS), an ongoing prospective study at Seoul National University Boramae Medical Center (Seoul, Korea). Written informed consent to participate was obtained from all of the subjects. The study protocol was approved by the Seoul National University Boramae Medical Center Institutional Review Board and conformed to the tenets of the Declaration of Helsinki.

All of the participants underwent a full ophthalmologic examination that included best-corrected visual acuity (BCVA) assessment, refraction, slit-lamp biomicroscopy, Goldmann applanation tonometry, gonioscopy, dilated funduscopic examination, keratometry (RKT-7700; Nidek, Hiroshi, Japan), axial length measurement (IOLMaster version 5; Carl Zeiss Meditec, Dublin, California, USA), disc photography and red-free fundus photography (TRC-NW8; Topcon, Tokyo, Japan), spectral-domain optical coherence tomography (SD-OCT; Spectralis OCT, Heidelberg Engineering, Heidelberg, Germany) and standard automated perimetry (Humphrey Field Analyzer II 750, 24–2 Swedish Interactive Threshold Algorithm; Carl-Zeiss Meditec, Dublin, CA, USA). During the acquisition of SD-OCT images, the subjects were asked to fixate on the target, and images were acquired with the forehead and chin stabilized by the headrest. Extra care was taken during each exam to confirm that the forehead and chin were correctly positioned and did not move. Before the initiation of treatment, IOP was measured repeatedly (typically 5 times) on the same or different days. From the documented IOP readings of each subject, the highest IOP was used for the subsequent analysis.

HTG was defined as glaucomatous optic nerve damage and associated visual field defects, an open iridocorneal angle, and a highest IOP > 21mmHg. Glaucomatous optic nerve damage was defined by rim thinning, notching and the presence of retinal nerve fiber layer (RNFL) defects, and was evaluated by a glaucoma specialist (SHK). Glaucomatous visual field defect was defined as (1) outside normal limits on glaucoma hemifield test, or (2) three abnormal points, with a *P* value less than 5% probability of being normal and one with a *P* value less than 1% by pattern deviation, or (3) pattern standard deviation of less than 5%. Visual field defects were confirmed on two consecutive reliable tests (fixation loss rate of ≤ 20%, and false-positive and false-negative error rates of ≤ 25%).

The inclusion criteria were HTG and myopia (axial length ≥ 24.0 mm) that had been followed up for at least 2 years. The exclusion criteria were, initial glaucoma diagnosis at age < 20 years old, BCVA of < 20/40, a sharply defined posterior staphyloma (which can deform the contour of the eyeball) on funduscopic examination, a history of ocular surgery other than cataract extraction or glaucoma surgery, retinal or neurologic disease other than glaucoma that could cause visual field defect, a poor-quality image (i.e., quality score <15) of any section on enhanced depth imaging (EDI) SD-OCT radial scans, and indeterminability of central vascular trunk position within the BMO. If both eyes were eligible, 1 eye was randomly selected as the study eye.

### Assessment of vascular trunk deviation

The peripapillary area was imaged by SD-OCT. The corneal curvature of each eye was entered into the SD-OCT system (Spectralis, Heidelberg Engineering) before performing SD-OCT scanning, so as to compensate for potential magnification error. The deep ONH complex was imaged using the EDI technique. The BMO was demarcated using the Glaucoma Module Premium Edition of the Spectralis machine. With 24 high-resolution radial scan images of the ONH, 15° apart from each other, each averaged from 24 individual B-scans, SD-OCT automatically detects the margin of the BMO. Every detected BMO margin was reviewed by one of the authors (KML), and errors were corrected manually. Based on the edited BMO margin, the Spectralis machine calculated the area and center of the BMO and determined the foveal-BMO axis.

**Fig 2:**
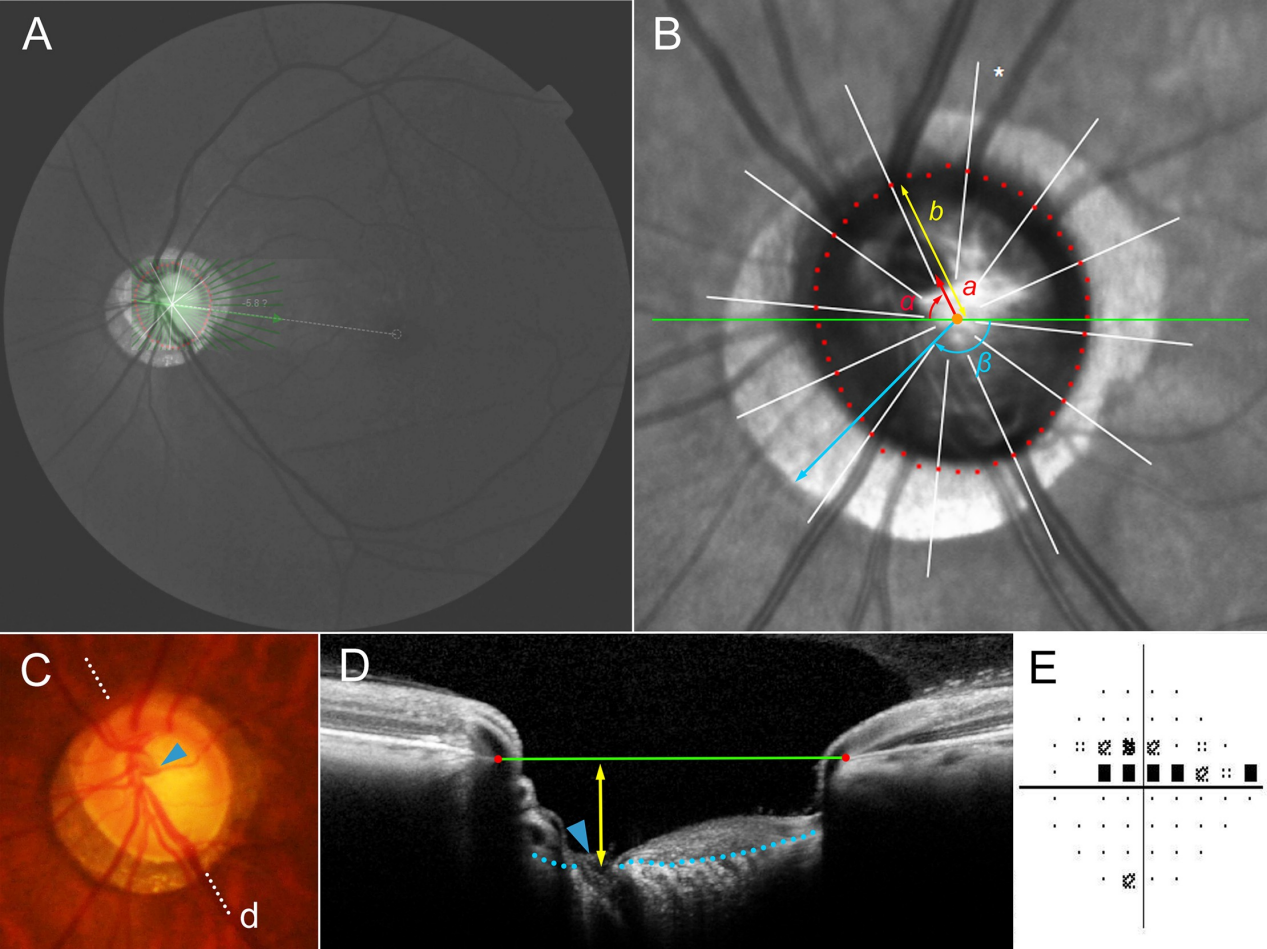
Measurement of position of central vascular trunk as surrogate of lamina cribrosa (LC) shift. (**A**) Red-free fundus photo. Diffuse retinal nerve fiber layer (RNFL) loss was observed in both hemispheres but was more severe in the inferior hemisphere. An infrared image obtained by spectral-domain optical coherence tomography (SD-OCT) is transposed to show the margin of the Bruch’s membrane opening (BMO) and the foveal-BMO axis. SD-OCT uses 24 radial scan images to delineate the BMO margin (red dots) and calculate the foveal-BMO axis. (**B**) Magnified view of peripapillary area. The red dots indicate the BMO margin, and the green line is the reference line. The angular deviation of the vascular trunk (α) is measured clock-wise, with the nasal horizontal midline as 0°. A positive value indicates the superior location, and a negative value indicates the inferior location relative to the reference line. From the BMO center, the distances are measured to the vascular trunk (*a*), and to the BMO margin in the same direction (*b*). The ratio of these distances is defined as the ‘shift index’ (*a*/*b*), which is used to measure the extent of shift. The angular location of maximum width of β-zone parapapillary atrophy (PPA) (β) is measured clock-wise from the reference line (green line), with the temporal horizontal midline as 0°. A positive value indicates the superior location and a negative value indicates the inferior location relative to the reference line. (**C**) Disc photograph. The dotted line indicates the location of the SD-OCT scan, targeted to the central retinal vascular trunk (arrowhead). (**D**) Cross-sectional image of SD-OCT clearly showing emergence of central retinal vascular trunk (arrowhead). The LC depth is measured as the vertical distance from the BMO plane (green reference line) to the vascular trunk (yellow double arrows). The blue-dotted line indicates the anterior LC surface margin. (**E**) The Humphrey visual field result shows scotoma only in the superior hemisphere.

The location of the central vascular trunk was demarcated on fundoscopic infrared images and color-disc photography (Fig 2). Its location was confirmed by cross-sectional SD-OCT imaging in all cases. In cases with an invisible vascular trunk on infrared fundus photographs and B-scan EDI SD-OCT images, fluorescein angiography or OCT angiography was used to determine the presence of the vascular trunk within the BMO. The position of central retinal vascular trunk was used as a surrogate of LC shift [11] since the vascular trunk of newborns was located mostly in the central area of the ONH [14]. The position of the central retinal vascular trunk was defined in two aspects: 1) its angular deviation (Fig 2B, α), and 2) the extent of shift (Fig 2B, *a*). The angle was measured based on the right-eye orientation, with the nasal horizontal midline as 0° (a positive value indicating a vascular trunk located superiorly, and a negative value indicating a vascular trunk located inferiorly). To evaluate the extent of shift, the distance of the vascular trunk from the center of the BMO (*a*) was divided by the distance of the BMO margin from the center of the BMO in that direction (*b*), and was defined ‘shift index’ (Fig 2B, *a*/*b*). In cases of invisible vascular trunk due to its being located outside the BMO, the shift index was defined as 1.0, and the angular deviations were not determined [11]. Using the Image J program (version 1.51, National Institutes of Health, Bethesda, Maryland, USA), one of authors (KML), who as blinded to the participants’ clinical information, measured the distances and angles. The reproducibility of the locating of the central vascular trunk was excellent, as we had stated in the previous study [11].

### Assessment of initial hemisphere developing visual field defect

The initial hemisphere developing visual field defect was determined retrospectively. Based on all of the obtained pattern deviation plots of each subject, three connected abnormal points with a *P* value less than 5% probability of being normal and at least one with a *P* value less than 1%, appearing repetitively in a single hemisphere and not in the other one, were classified as initial visual field defect in that hemisphere (Fig 2E). If those connected abnormal points were observed in both hemispheres, even at the initial examination, the eye was classified as bi-hemispheric involvement.

### Assessment of ONH structure

The optic disc and PPA parameters were measured on the same infrared fundus images by one observer (KML) blinded to the subjects’ information. The LC depth (LCD) was defined as the linear distance of the central retinal vascular trunk from the BMO plane, and it was measured using the built-in caliper tool of the Spectralis viewer (Fig 2D).

β-zone parapapillary atrophy (PPA) was defined as the area without any retinal pigment epithelium (RPE) adjacent to the ONH [10]. The angular location of maximal width of β-zone PPA was measured from the BMO center (Fig 2B, β) in every eye that had β-zone PPA. The angle was measured based on the right-eye orientation, with the temporal horizontal midline as 0° (a positive value indicating a superior angular location, and a negative value indicating an inferior angular location) [11]. Measurements were performed using the Image J program (version 1.51, National Institutes of Health) in the same manner as measured for the angular deviation of central retinal vascular trunk.

### Data analysis

The group comparisons were performed by ANOVA test, with the post hoc Scheffe test for the continuous variables and the chi-square test for the categorical variables. Logistic regression analysis was used to reveal the risk factors determining the initial hemisphere of visual field defect. Conditional inference tree analysis was used to reveal the hierarchy of risk factors. This recursive partitioning method allows for unbiased testing of categorical as well as continuous variables without any statistical assumptions [15]. Statistical analyses were performed with commercially available software (Stata version 14.0; StataCorp, College Station, Texas, USA) and R statistical packages version 3.4.3 (available at http://www.R-project.org; assessed December 5, 2017). The data herein are presented as the mean±standard deviation except where stated otherwise, and the cutoff for statistical significance was set at *P*<0.05.

## Results

This study initially involved 92 HTG patients with myopia. Of these, 3 patients were excluded due to poor image quality of radial scans leading to incomplete visualization of the BMO margin, 2 patients due to bifurcation of the central vascular trunk, and 3 patients due to combined retinal abnormalities. Vascular trunk emergence was not evident in 9 patients on infrared imaging. Among these, 6 patients were proved to not have the vascular trunk within the BMO by angiography, while 3 patients who had declined the angiography were excluded, leaving a final sample of 81 HTG patients with myopia (70 primary open-angle glaucoma, 8 steroid-induced glaucoma, and 3 angle-recession glaucoma). The subjects were aged 54.7±14.2 years (females: 25 [31%]), had a refractive error of –3.17±3.04 *D*, a highest IOP of 25.2±4.1 (range 22–42) mmHg, an axial length of 25.5±1.3 (range 24.03–30.89) mm, an initial mean deviation of –8.11±5.85 *dB*, and a total follow-up period of 5.0±2.7 years.

At the initial visits, 36 eyes (44%) had superior visual field defects, 27 (33%) inferior visual field defects, and 18 (22%) bi-hemispheric visual field defects. At the final visits, 27 eyes (33%) had superior visual field defects, 20 (25%) inferior visual field defects, and 34 (42%) bi-hemispheric visual field defects.

The patients were classified into three groups according to the shift index: 1) group A with shift index <0.5 (mild shift); 2) group B with shift index ≥0.5 and <1 (moderate shift); and 3) group C with shift index = 1 (severe shift) (Table 1). Greater shift index was related to younger age and longer axial length. LCD, measured at the point of the vascular trunk, did not differ between the mild and moderate shift group. At the initial visits, bi-hemispheric involvement was observed in 7 eyes (18%) in the mild shift group, 7 (19%) in the moderate shift group, and 4 (66%) in the severe shift group. At the final visits, however, more eyes had developed bi-hemispheric visual field defects: 15 (39%) in the mild shift group, 15 (41%) in the moderate shift group, and 4 (66%) in the severe shift group (Table 1). The mean follow-up periods did not differ among the groups (Table 1).

**Table 1:** Demographic data according to shift index.

	Mild shift group (A) Shift Index<0.5 (N = 38)	Moderate shift group (B) 0.5≤Shift Index<1 (N = 37)	Severe shift group (C) Shift Index = 1 (N = 6)	*P*	Post Hoc Test
Age, *years*	60.3±11.6	51.5±14.6	38.8±8.8	<0.001*	A>B = C
Sex (Male / Female)	29 / 9	23 / 14	4 / 2	0.411^†^	
Highest IOP, *mmHg*	25.6±5.0 (range 22–42)	25.1±3.2 (range 22–34)	23.8±2.9 (range 22–28)	0.618*	
Axial length, *mm*	25.0±1.0 (range 24.03–27.7)	25.9±1.5 (range 24.05–30.89)	26.4±1.0 (range 25.43–27.73)	0.003*	A<B = C
Mean deviation at initial visit, *dB*	-8.37±5.86	-7.80±6.05	-8.41±5.33	0.910*	
Mean deviation at final visit, *dB*	-10.90±6.58	-10.31±6.57	-9.65±7.52	0.875*	
Follow-up period, years	5.1±2.7	5.1±2.8	3.3±1.8	0.295	
Bi-hemispheric visual field defects at initial visits	7 (18%)	7 (19%)	4 (66%)	<0.025^†^	
Bi-hemispheric visual field defects at final visits	15 (39%)	15 (41%)	4 (66%)	0.442^†^	
Bi-hemispheric RNFL defects at final visits	29 (76%)	30 (81%)	6 (100%)	0.394^†^	
BMO area, *mm*^*2*^	2.42±0.56	2.90±1.23	2.65±0.70	0.091*	
Shift Index	0.25±0.14	0.68±0.12	1.0	<0.001*	A<B<C
Angular deviation of vascular trunk,*°* (absolute value)	37.7±42.8	30.8±44.1	NA	0.490^‡^	
Angular location of maximal width of β-zone PPA, *°*	-11.9±58.2	-32.9±53.4	-6.5±16.9	0.210*	
LCD measured at the point of vascular trunk, *μm*	546±150	531±165	NA	0.671^‡^	
Secondary glaucoma^¶^	7 (16%)	4 (13%)	0	0.600^†^	

IOP = intraocular pressure; RNFL = retinal nerve fiber layer; BMO = Bruch’s membrane opening; NA = not applicable; PPA = parapapillary atrophy; LC = lamina cribrosa; LCD = LC depth

* Comparison performed using ANOVA test with post hoc Scheffe test to compare differences among three groups

^†^Comparison performed using Chi-square test

^‡^Comparison performed using independent-*t* test

^¶^ Group A: 4 eyes with steroid-induced glaucoma and 3 eyes with angle-recession glaucoma; Group B: 4 eyes with steroid-induced glaucoma

**Fig 3:**
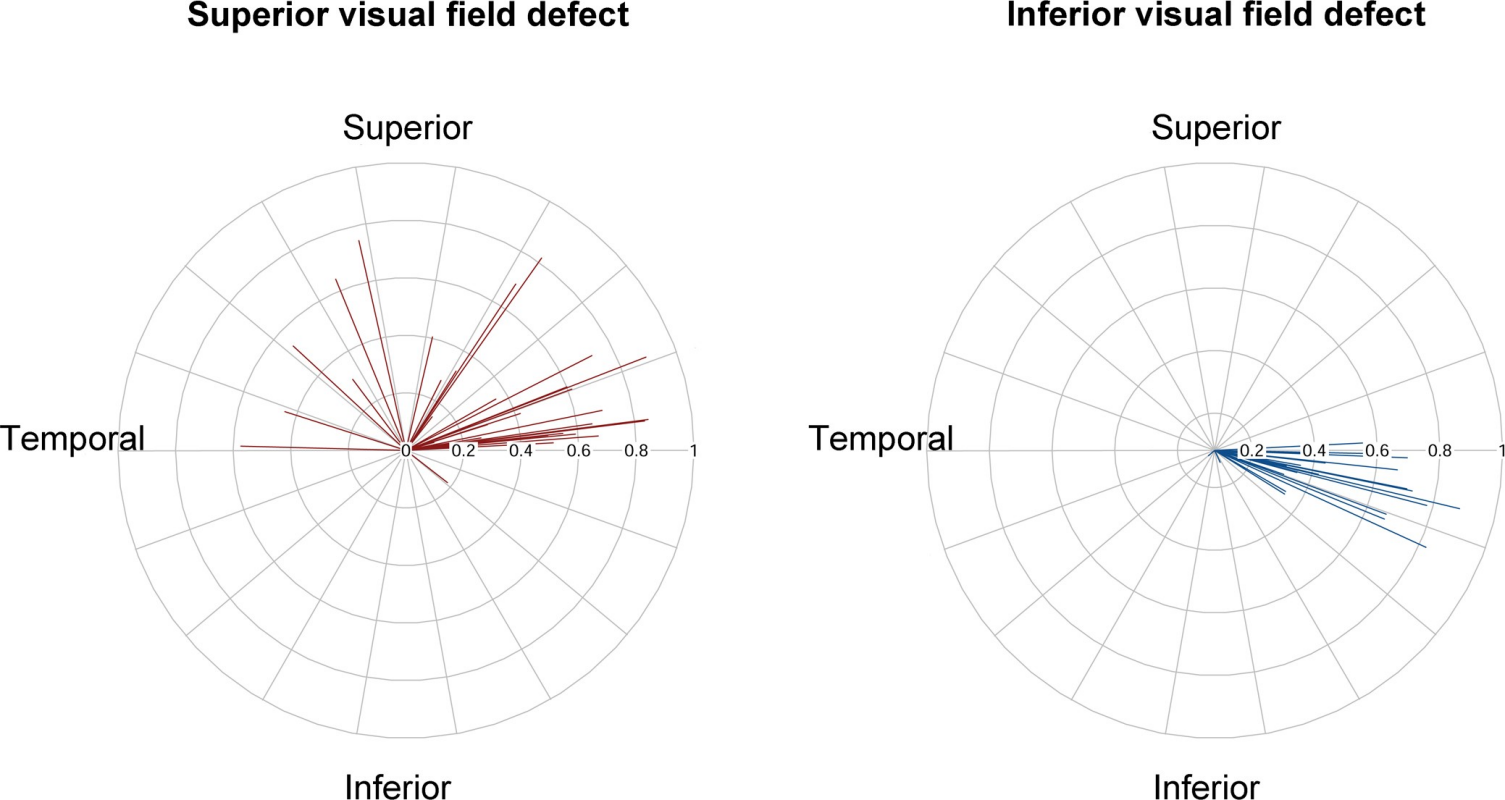
Angular deviation of vascular trunk from Bruch’s membrane opening (BMO) center according to initial superior visual field defect or inferior visual field defect. The polar plots demonstrate the deviation of the vascular trunk (red lines: superior visual field defect group, blue lines: inferior visual field defect group). The data on the left eyes are flipped to the right eye orientation. The shift index of each subject is incorporated into the same length of both red and blue lines. Eyes with severe shift (shift index = 1.0) were excluded, because in those cases, we could not determine the location of the vascular trunk.

**Fig 4:**
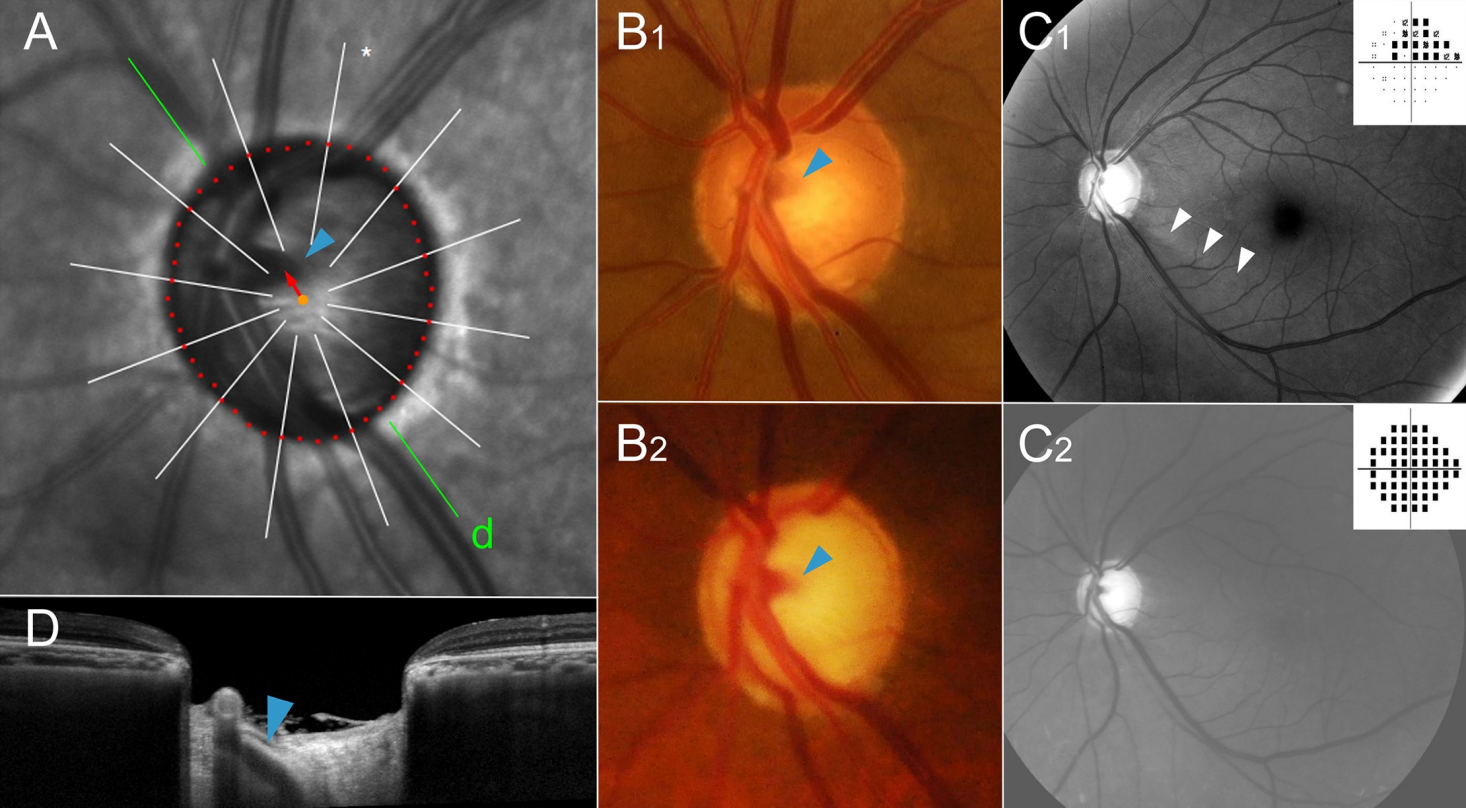
Representative case of initial superior visual field defect group. This patient (66 year-old, male) has been followed up for 9 years. Highest intraocular pressure on his left eye was 36 mmHg and he had underwent trabeculectomy in 8 years ago. His compliance to the medication was not good and he still refuses the second operation despite the continued glaucoma progression. (**A**) Magnified view of peripapillary area of infrared funduscopic image. The red dots indicate the Bruch’s membrane opening (BMO), which was delineated using cross-sectional B-scan spectral-domain optical coherence tomography (SD-OCT) images. Please note the superior vascular trunk (blue arrowhead) deviation (red arrow) from the BMO center (orange dot). The green line indicates the location of the SD-OCT scan. (**B**) Disc photographs, (**C**) red-free fundus photos and pattern deviation plots of Humphrey visual field tests at initial (**B**_**1**_ & **C**_**1**_) and final (**B**_**2**_ & **C**_**2**_) visits. Please note that the position of the vascular trunk had not been changed in the course of disease progression (**B**_**1**_ and **B**_**2**_ blue arrowheads). The retinal nerve fiber layer (RNFL) defect had been observed on the inferior side only at the initial visit (**C**_**1**_, white arrowheads), while it had progressed to diffuse atrophy in both hemispheres at the final visit (**C**_**2**_). (**D**) Emergence of the central vascular trunk is evident (blue arrowhead).

**Fig 5:**
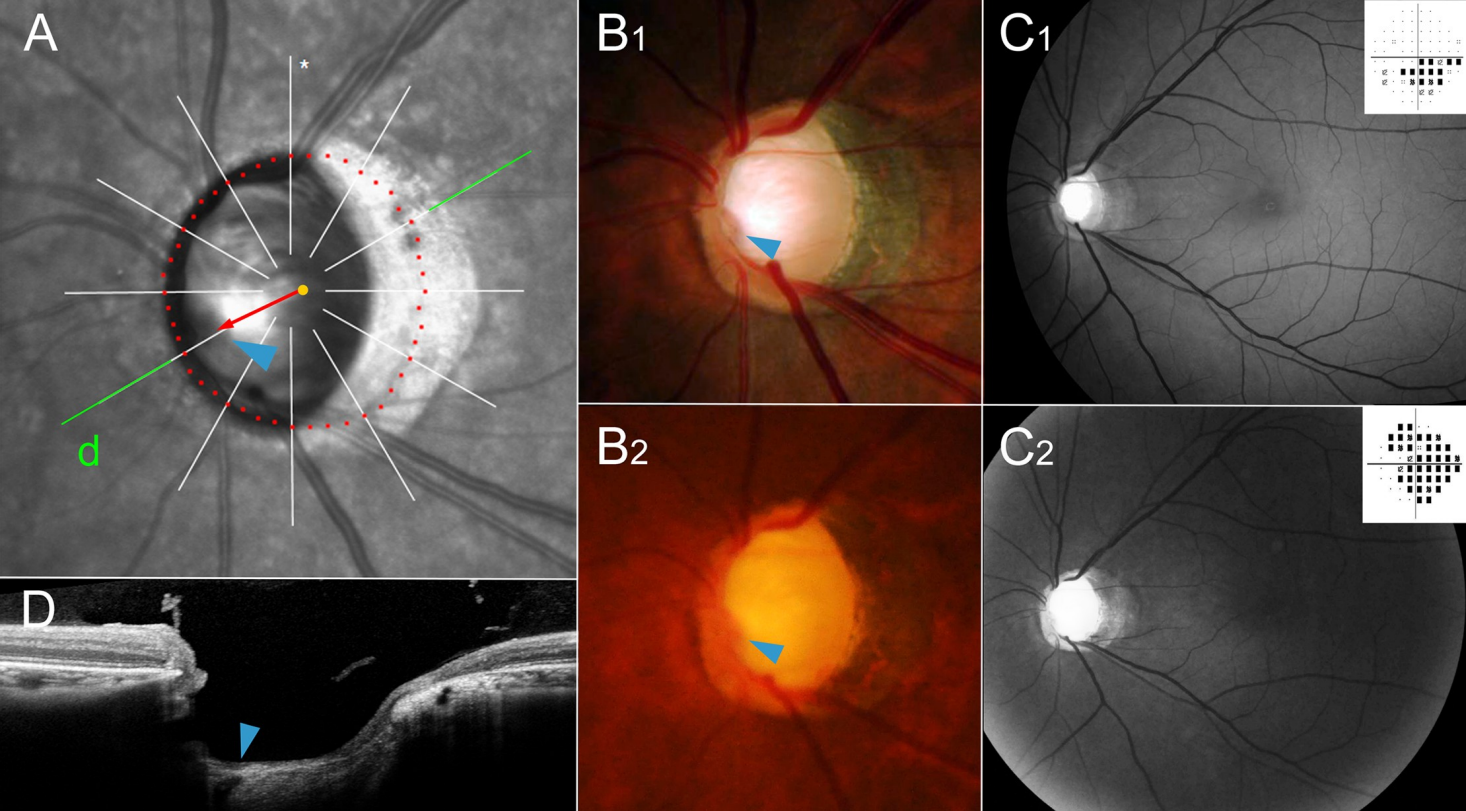
Representative case of initial inferior visual field defect group. This patient (40 year-old, male) has been followed up for 14 years. Highest intraocular pressure on his left eye was 25 mmHg. He did not use any glaucoma medication for 3 years while he was working abroad. (**A**) Magnified view of peripapillary area of infrared funduscopic image. The red dots indicate the Bruch’s membrane opening (BMO), which was delineated using cross-sectional B-scan spectral-domain optical coherence tomography (SD-OCT) images. Please note the inferior vascular trunk (blue arrowhead) deviation (red arrow) from the BMO center (orange dot). The green line indicates the location of the SD-OCT scan. (**B**) Disc photographs, (**C**) red-free fundus photos and pattern deviation plots of Humphrey visual field tests at initial (**B**_**1**_ & **C**_**1**_) and final (**B**_**2**_ & **C**_**2**_) visits. Please note that the position of the vascular trunk had not been changed in the course of disease progression (**B**_**1**_ and **B**_**2**_ blue arrowheads). The retinal nerve fiber layer (RNFL) defect had been observed as diffuse atrophy, which was more severe in the superior hemisphere at the initial visit (**C**_**1**_), whereas it had progressed in both hemispheres at the final visit (**C**_**2**_). (**D**) Emergence of the central vascular trunk is evident (blue arrowhead).

**Fig 6:**
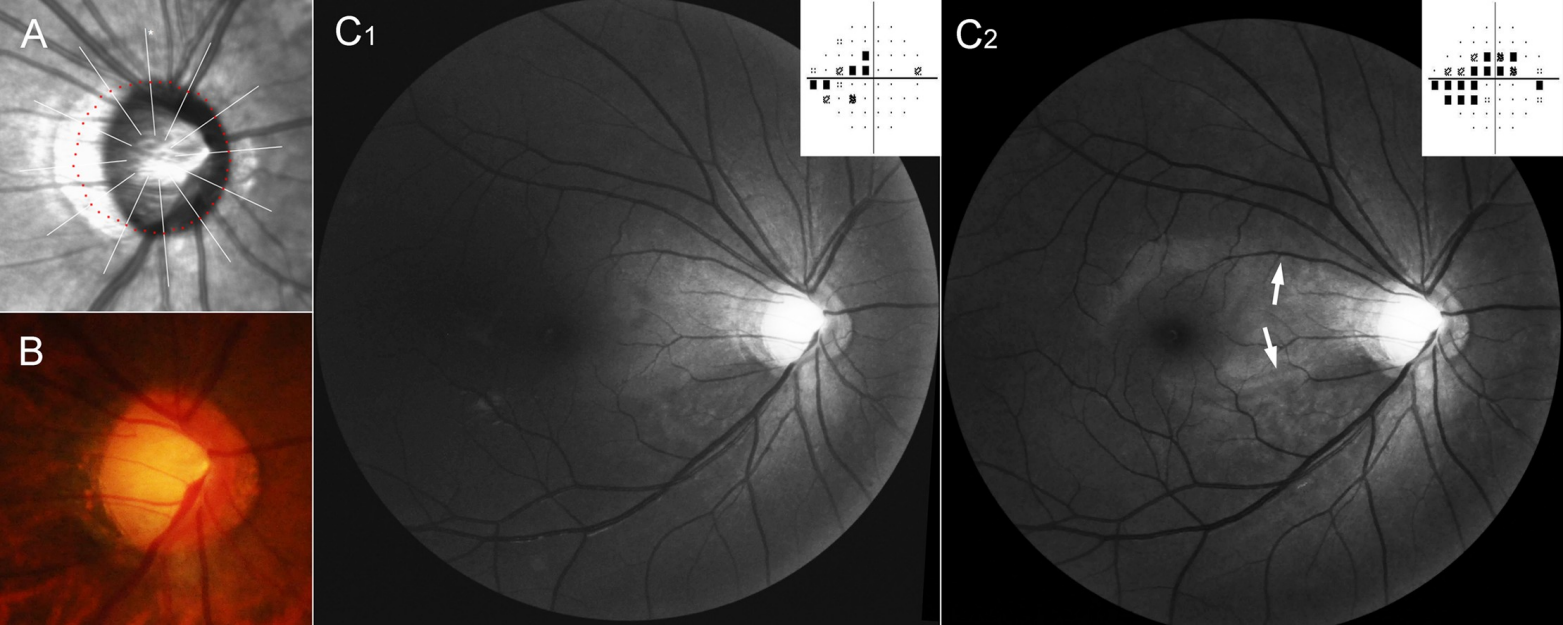
Representative case of initial bi-hemispheric visual field defects in severe shift group. This patient (40 year-old, female) has been followed up for 6 years. Highest intraocular pressure on her right eye was 23 mmHg. (**A**) Magnified view of peripapillary area of infrared funduscopic image. The red dots indicate the Bruch’s membrane opening (BMO), which was delineated using cross-sectional B-scan spectral-domain optical coherence tomography (SD-OCT) images. (**B**) Disc photography. The emergence of the vascular trunk is not evident. (**C**) Red-free fundus photos and pattern deviation plots of Humphrey visual field tests at initial (**C**_**1**_) and final (**C**_**2**_) visits. The bi-hemispheric visual field defects and bi-hemispheric retinal nerve fiber layer (RNFL) defects were observed at the initial visit (**C**_**1**_). At the final visit, the RNFL defects had increased toward macula (arrows). The visual field defect had also progressed in both hemispheres (**C**_**2**_).

The initial hemisphere with visual field defect was determined by the angular deviation of the vascular trunk position (Fig 3). The initial superior visual field defect group had superiorly displaced vascular trunks (Fig 4), while the initial inferior visual field defect group had inferiorly displaced vascular trunks (Fig 5). The eyes in the severe shift group tended to have bi-hemispheric visual field defects from the initial visits (Fig 6). The logistic regression analysis revealed that the inferior deviation of the vascular trunk was the only risk factor for earlier involvement of visual field defect in the inferior hemisphere (*P* = 0.001, Table 2).

**Table 2:** Risk factors of initial visual field defect developing in inferior hemisphere.

	Univariate analysis			Multivariate analysis*		
	OR	95% CI	*P*	OR	95% CI	*P*
Age, *years*	1.028	(0.991 to 1.066)	0.136	1.033	(0.957 to 1.114)	0.404
Highest IOP, *mmHg*	0.996	(0.873 to 1.135)	0.947			
Axial length, *mm*	0.961	(0.666 to 1.386)	0.831			
BMO area, *mm*^*2*^	1.188	(0.725 to 1.947)	0.493			
Mean deviation at initial visit, *dB*	1.051	(0.944 to 1.169)	0.365			
LCD, *μm*	0.999	(0.996 to 1.002)	0.596			
Angular location of β-zone PPA, *°*	1.007	(0.997 to 1.017)	0.197	0.983	(0.961 to 1.006)	0.136
Shift index	0.391	(0.052 to 2.923)	0.360			
Angular deviation of vascular trunk, *°*	**0.830**	**(0.746 to 0.922)**	**0.001**	**0.818**	**(0.732 to 0.918)**	**0.001**

OR = odds ratio; CI = confidence interval; IOP = intraocular pressure; BMO = Bruch’s membrane opening; PPA = parapapillary atrophy; LCD = lamina cribrosa depth

Statistically significant values (*P*<0.05) are shown in bold.

*Variables with *P*<0.20 in the univariate analysis were included in the subsequent multivariate analysis. Angular location of β-zone PPA was measured at the point where each PPA was at the maximal distance from the BMO center.

**Fig 7:**
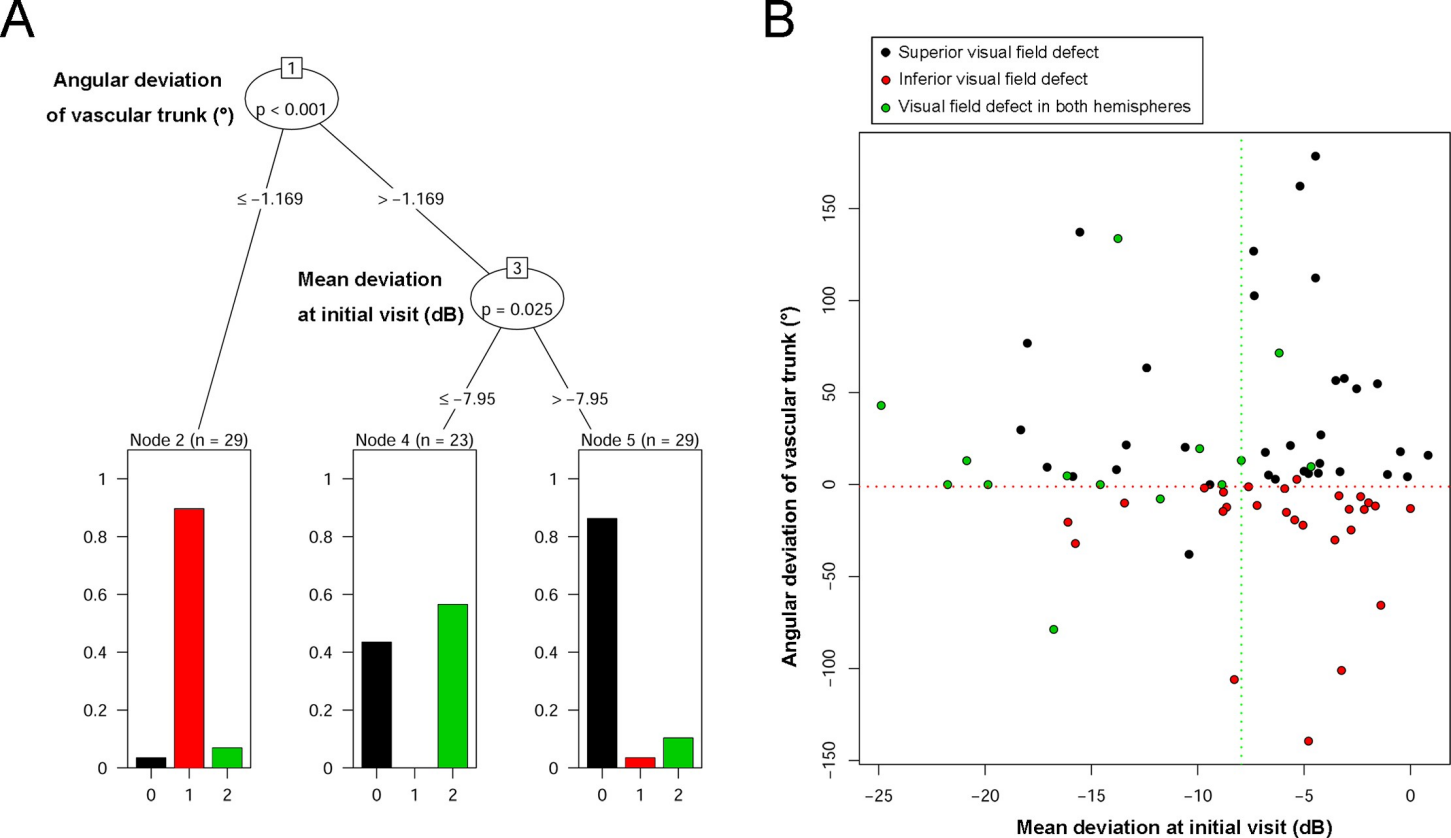
Conditional inference tree analysis. (**A**) By a recursive partitioning method, this analysis allows for unbiased testing of both categorical and continuous variables without any statistical assumptions. By this analysis, the initial hemisphere of visual field defect was categorized into three terminal nodes. First, the angular deviation of the vascular trunk was associated with the initial hemisphere of visual field defect in the upper level: superior vascular trunk deviation was associated with initial superior visual field defect, while inferior vascular trunk deviation was associated with initial inferior visual field defect. Then, bi-hemispheric involvement was associated with worse mean deviation at the initial visits in the lower level. Subjects with a delayed diagnosis would present a more progressed disease status with bi-hemispheric visual field defects, since the high intraocular pressure would eventually affect all of the areas of the LC. (**B**) A scatter plot showing the effects of angular deviation of vascular trunk and initial mean deviation on the initial hemisphere of visual field defect. Black indicates subjects with initial superior visual field defect, red indicates subjects with initial inferior visual field defect, and green indicates subjects with initial bi-hemispheric visual field defects. A red dashed line is drawn along an angular deviation of vascular trunk of -1.169°, and a green dashed line is drawn along a mean deviation of -7.95 *dB* to visualize the distribution of each group according to these two variables. Please note that the eyes with severe shift (shift index = 1.0) are not drawn in the scatter plot, since in those cases, we could not locate the angular deviation of vascular trunk.

At the initial visits, initial bi-hemispheric visual field defects were associated with worse mean deviation (*P*<0.001, Table 3). On the other hand, at the final visits, visual field defects restricted to a single hemisphere were associated with better mean deviation (*P*<0.001), closer angular deviation of vascular trunk to the vertical axis (*P* = 0.017), and lesser highest IOP with marginal significance (*P* = 0.075, Table 4). The conditional inference tree analysis results summarized those relationships described above: the initial hemisphere of visual field defect was associated with the angular deviation of vascular trunk in the upper level, and with the initial value of mean deviation in the lower level (Fig 7A). The visual field defect occurred in the same hemisphere as did vascular trunk deviation, while the eyes with worse mean deviation from their initial visits had a tendency of bi-hemispheric visual field defect (Fig 7B).

**Table 3:** Risk factors of bi-hemispheric visual field defects at initial visit.

	Univariate analysis		
	OR	95% CI	*P*
Age, *years*	0.993	(0.957 to 1.031)	0.724
Highest IOP, *mmHg*	1.022	(0.904 to 1.156)	0.723
Axial length, *mm*	0.893	(0.584 to 1.367)	0.604
BMO area, *mm*^*2*^	0.867	(0.463 to 1.623)	0.655
Mean deviation at initial visit, *dB*	**0.817**	**(0.735 to 0.909)**	**<0.001**
LCD, *μm*	1.001	(0.997 to 1.005)	0.654
Shift index	2.284	(0.342 to 15.268)	0.394
Angular deviation of vascular trunk, *°* (absolute value)	0.995	(0.980 to 1.011)	0.559

OR = odds ratio; CI = confidence interval; IOP = intraocular pressure; BMO = Bruch’s membrane opening; PPA = parapapillary atrophy; LCD = lamina cribrosa depth

Statistically significant values (*P*<0.05) are shown in bold. Except for the mean deviation, all of the variables had a P value larger than 0.20. Therefore, multivariate analysis was not performed.

**Table 4:** Risk factors of visual field defect restricted to single hemisphere at final visit.

	Univariate analysis			Multivariate analysis*		
	OR	95% CI	*P*	OR	95% CI	*P*
Age, *years*	0.996	(0.965 to 1.027)	0.786			
Highest IOP, *mmHg*	0.899	(0.802 to 1.009)	0.070	0.854	(0.718 to 1.016)	0.075
Axial length, *mm*	1.040	(0.740 to 1.460)	0.823			
BMO area, *mm*^*2*^	1.137	(0.697 to 1.856)	0.607			
Mean deviation at final visit, *dB*	**1.271**	**(1.143 to 1.413)**	**<0.001**	**1.290**	**(1.136 to 1.465)**	**<0.001**
LCD, *μm*	0.998	(0.995 to 1.001)	0.148	0.997	(0.993, 1.001)	0.157
Shift index	0.947	(0.195 to 4.602)	0.947			
Obliqueness of vascular trunk deviation, *°*	**1.022**	**(0.999 to 1.046)**	**0.061**	**1.042**	**(1.007 to 1.079)**	**0.017**
Follow-up period, years	1.006	(0.854, 1.186)	0.942			

OR = odds ratio; CI = confidence interval; IOP = intraocular pressure; BMO = Bruch’s membrane opening; PPA = parapapillary atrophy; LCD = lamina cribrosa depth

Statistically significant values (*P*<0.05) are shown in bold.

*Variables with *P*<0.20 in the univariate analysis were included in the subsequent multivariate analysis. Obliqueness of vascular trunk deviation was defined as the absolute value of angular deviation in the quadrant where the vascular trunk was located in order to represent the extent to which the vascular trunk deviated from the vertical axis.

## Discussion

In the present study, we investigated whether the position of the central vascular trunk, as a surrogate of LC shift, was associated with the initial hemispheric location of glaucomatous visual field defect in myopic HTG. Superior deviation of vascular trunk was associated with initial visual field defects in the superior hemisphere, while inferior deviation of vascular trunk was associated with initial visual field defects in the inferior hemisphere. Both hemispheres gradually became involved as glaucoma progressed. This implied that the counter hemisphere of the LC shift might be more vulnerable to glaucomatous damage driven by high IOP.

In our Boramae Myopia Cohort Study, we found that the inner retinal structures including the BMO were preserved whereas the outer load-bearing structures were shifted by relative overgrowth during axial elongation [8–10]. This implies that the LC, as a part of the outer wall, would also be shifted [9]. This speculation was supported by the positional change of the central vascular trunk, which is embedded in the dense connective tissue of the LC [9]. Since the vascular trunk of newborns was located mostly in the central area of the ONH [14], vascular trunk deviation within the BMO could represent LC shift relative to the inner retinal structures, which were acquired during growth. In myopic NTG, the direction and amount of LC shift were closely associated with the angular location of RNFL defects: the RNFL defect occurred in the direction opposite to the LC shift, while unbiased shifting between hemispheres resulted in severe shift and bi-hemispheric RNFL defects [11]. Thus, LC shift could be a source of tensile stress that makes the ONH vulnerable to glaucomatous damage even in cases of normal IOP.

Glaucomatous optic neuropathy has preferential locations of damage: superotemporal and inferotemporal regions [4–6]. This fact is attributed to the regional pore-size differences of the LC: the superior and inferior pores are larger than the others [4, 6]. As we stated above, LC shift would add additional tensile stress to those susceptible pores in the direction opposite to the shift. In NTG eyes, the stress is confined to the side opposite to the shift, the other hemisphere being spared [11, 16]. In the presence of high IOP, however, radical even-handed stress is applied to all pores of the LC regardless of whether they are located in the shifted or counter side of the LC. Therefore, the axons within the more vulnerable pores (on the counter side of the shift) would be damaged initially, though all of them would be damaged eventually (Fig 1).

The frequent types of optic nerve damage and RNFL defect have been reported to be diffuse in HTG and localized in NTG [17–21]. On the contrary, others have found similar patterns of optic nerve damage for HTG and NTG, which would suggest that they are both on the same disease spectrum [22–25]. Our data favored the former: the effect of high IOP was universal, and eventually resulted in diffuse atrophy of both hemispheres. This was supported by the result of our study, which is that most of the HTG eyes (80%) had bi-hemispheric RNFL defects in the end (Table 1). When assessed by SD-OCT, more eyes (84%) had RNFL thinning from their age-matched normative database in both hemispheres, which was suggestive of the hidden diffuse nature of glaucomatous damage in HTG eyes. Nevertheless, we also found a common point between HTG and NTG: the hemisphere opposite to shifting was earlier affected by glaucomatous optic neuropathy. Considering all of the data together, it is clear that the location of optic nerve damage is dependent on both IOP and LC shift, which could explain both the difference and the similarity between HTG and NTG.

Initial bi-hemispheric visual field defects were associated with worse mean deviation at the initial visits. This indicated a more advanced disease status. Since IOP affects all LC pores, bi-hemispheric defects would be observed in cases where the subject was given a delayed diagnosis of a more advanced stage of HTG. Similarly, the visual field defects restricted to a single hemisphere at the final visits were associated with better mean deviation. Moreover, more vertical LC shift was associated with visual field asymmetry at the final visits. Vertical LC shift would generate marked asymmetry between the LC’s superior and inferior pores, thereby resulting in single-hemispheric visual field defects.

Subjects in the moderate and severe shift groups were younger and had longer axial length than subjects in the mild shift group. We speculated that the larger shift in the former was associated with the longer axial length. The association of younger age and larger axial length can be understood in two ways. On the one hand, there is a global trend of increasing myopia [26]; therefore, including more young subjects would mean having more subjects with myopia and with a larger shift index. On the other hand, larger shift might be associated with greater tensile stress that would incur glaucomatous optic nerve damage at younger ages. Further study will be necessary to address this issue.

Interestingly, the vascular trunk positions were spread more widely in the superior visual field defect group than in the inferior visual field defect group. This may be attributable to two factors. First, superior LC shift was more frequent than inferior LC shift in our study group, and thus, the former had a larger variance than the latter had. Second, we could not observe as much inferotemporal shift as superotemporal shift. We speculated that the regional difference of scleral properties might result in asymmetric expansion between the superior and inferior walls, thereby limiting the inferotemporal shift of the vascular trunk. Further study will be necessary to elucidate the cause and implication of this asymmetry of shift direction.

This study has several limitations. First, the study design was retrospective. Treatment had differed among subjects and was not standardized. Therefore, we could not compare the progression rates between the groups. This notwithstanding, we evaluated only the sequence of hemispheric visual field defects in HTG, which, we believe, would not be affected by treatment non-standardization. Further, longitudinal study would be required to address the effect of LC shift on glaucoma progression. Second, we compared the vascular trunk position with the BMO center in determining LC shift, despite the fact that we did not observe LC shift in real time. Most of newborns, however, had the centrally located vascular trunk position [14]. Further, actual LC shift was demonstrated in our previous prospective cohort study [8–10]. Therefore, the vascular trunk deviation could be used as a surrogate of LC shift. Finally, we did not analyze the association between the location of RNFL defect and LC shift in this study, because most of the HTG eyes had diffuse RNFL defect, in which cases, demarcation of the defect was not possible.

In conclusion, high IOP in myopic eyes affected the axons within the superior or inferior hemisphere counter to LC shift first, and then, gradually both hemispheres. This sequential involvement supports the notion that LC shift renders the LC more vulnerable to glaucomatous damage, especially the pores on the side counter to the shift.
